# On-treatment biopsies to predict response to neoadjuvant chemotherapy for breast cancer

**DOI:** 10.1186/s13058-024-01883-w

**Published:** 2024-09-24

**Authors:** Bruno Valentin Sinn, Katharina Sychra, Michael Untch, Thomas Karn, Marion van Mackelenbergh, Jens Huober, Wolfgang Schmitt, Frederik Marmé, Christian Schem, Christine Solbach, Elmar Stickeler, Hans Tesch, Peter A. Fasching, Andreas Schneeweiss, Volkmar Müller, Johannes Holtschmidt, Valentina Nekljudova, Sibylle Loibl, Carsten Denkert

**Affiliations:** 1 Department of Pathology, Charité – Universitätsmedizin Berlin, 10117 Berlin, Germany; 2 Department of Gynecology, Helios Kliniken Berlin-Buch, Berlin, Germany; 3 Department of Gynecology and Obstetrics, Goethe-University, Frankfurt, Germany; 4 Department of Gynecology and Obstetrics, Universitätsklinikum Schleswig-Holstein, Kiel, Germany; 5 Breast Center St. Gallen, Kantonsspital St. Gallen, St. Gallen, Switzerland; 6 Department of Gynecologic Oncology, Medical Faculty Mannheim, Heidelberg University, University Hospital Mannheim, Mannheim, Germany; 7 Mammazentrum Hamburg, Hamburg, Germany; 8 Breast Center, Universitätsklinikum Frankfurt, Frankfurt, Germany; 9 Department of Gynecology, Uniklinik RWTH Aachen, Aachen, Germany; 10 Centrum für Hämatologie und Onkologie Bethanien, Frankfurt am Main, Germany; 11 Department of Gynecology and Obstetrics, University Hospital Erlangen, Erlangen, Germany; 12 Universitätsfrauenklinik Heidelberg, Heidelberg, Germany; 13 Department of Gynecology, Universitätsklinikum Hamburg-Eppendorf, Hamburg, Germany; 14 German Breast Group, Neu-Isenburg, Germany; 15 Department of Pathology, Philipps-University Marburg and University Hospital Marburg (UKGM), Marburg, Germany; 16 UCT-University Cancer Center, Frankfurt-Marburg, Germany

**Keywords:** Breast cancer, Neoadjuvant therapy, Serial biopsies, TILs, Ki-67

## Abstract

**Background:**

Patients with pathologic complete response (pCR) to neoadjuvant chemotherapy for invasive breast cancer (BC) have better outcomes, potentially warranting less extensive surgical and systemic treatments. Early prediction of treatment response could aid in adapting therapies.

**Methods:**

On-treatment biopsies from 297 patients with invasive BC in three randomized, prospective neoadjuvant trials were assessed (GeparQuattro, GeparQuinto, GeparSixto). BC quantity, tumor-infiltrating lymphocytes (TILs), and the proliferation marker Ki-67 were compared to pre-treatment samples. The study investigated the correlation between residual cancer, changes in Ki-67 and TILs, and their impact on pathologic complete response (pCR) and disease-free survival (DFS).

**Results:**

Among the 297 samples, 138 (46%) were hormone receptor-positive (HR+)/human epidermal growth factor 2-negative (HER2−), 87 (29%) were triple-negative (TNBC), and 72 (24%) were HER2+. Invasive tumor cells were found in 70% of on-treatment biopsies, with varying rates across subtypes (HR+/HER2−: 84%, TNBC: 62%, HER2+: 51%; *p* < 0.001). Patients with residual tumor on-treatment had an 8% pCR rate post-treatment (HR+/HER2−: 3%, TNBC: 19%, HER2+: 11%), while those without any invasive tumor had a 50% pCR rate (HR+/HER2−: 27%; TNBC: 48%, HER2+: 66%). Sensitivity for predicting residual disease was 0.81, with positive and negative predictive values of 0.92 and 0.50, respectively. Increasing TILs from baseline to on-treatment biopsy (if residual tumor was present) were linked to higher pCR likelihood in the overall cohort (OR 1.034, 95% CI 1.013–1.056 per % increase; *p* = 0.001) and with a longer DFS in TNBC (HR 0.980, 95% CI 0.963–0.997 per % increase; *p* = 0.026). Persisting or increased Ki-67 was associated with with lower pCR probability in the overall cohort (OR 0.957, 95% CI 0.928–0.986; *p* = 0.004) and shorter DFS in TNBC (HR 1.023, 95% CI 1.001–1.047; *p* = 0.04).

**Conclusion:**

On-treatment biopsies can predict patients unlikely to achieve pCR post-therapy. This could facilitate therapy adjustments for TNBC or HER2 + BC. They also might offer insights into therapy resistance mechanisms. Future research should explore whether standardized or expanded sampling enhances the accuracy of on-treatment biopsy procedures.

*Trial registration* GeparQuattro (EudraCT 2005-001546-17), GeparQuinto (EudraCT 2006-005834-19) and GeparSixto (EudraCT 2011-000553-23).

**Supplementary Information:**

The online version contains supplementary material available at 10.1186/s13058-024-01883-w.

## Introduction

Retrospective analyses of prospective breast cancer (BC) trials have shown comparable efficacy between chemotherapy administered in the adjuvant versus neoadjuvant settings [1]. Excellent response, defined as achieving pathologic complete response (pCR) to neoadjuvant chemotherapy (NACT), varies across breast cancer (BC) subtypes and is strongly influenced by the treatment regimen used. During the analyzed trials, pCR rates were approximately 50% and 30% for patients with triple-negative and HER2-positive disease, respectively [2]. In recent developments, incorporating immune checkpoint inhibitors into NACT for TNBC resulted in pCR rates of 64.8% [3], while employing dual anti-HER2 blockade yielded pCR rates of 66.2% in HER2-positive disease, depending on hormone receptor status [4]. Excellent response serves as a prognostic indicator for patient survival, especially in triple-negative and HER2-positive disease [5], and neoadjuvant therapy can serve as an in vivo assay for chemotherapy response.

Prediction of therapy response is of clinical interest offering the potential to customize treatment approaches and enhance response rates. With the emergence of new therapies and refined treatment protocols, there arises the question of whether de-escalating local and/or systemic treatment is viable for patients with a strong likelihood of achieving pCR [6]. For example, PET-based imaging can be used to predict pCR in patients with HER2-positive BC during neoadjuvant treatment with dual anti-HER2 treatment [7].

The conventional method for identifying response markers involves correlating genomic measurements from pre-therapeutic samples with clinical outcomes [8]. On-treatment tissue samples offer the opportunity for microscopic confirmation of response, biomarker examination, and tissue provision for translational research. However, the reliability of biopsy procedures and histopathological assessment for predicting on-treatment pCR remains uncertain. The RESPONDER trial examines if vacuum-assisted biopsies can be used to predict response in the breast with a false negative rate below 10% [9].

In neoadjuvant aromatase inhibition for HR + BC, gene expression analysis of on-treatment samples has been shown to predict treatment response and patient survival [10]. Ki-67 immunohistochemistry can indicate the need to switch to neoadjuvant chemotherapy if Ki-67 levels remain elevated during endocrine treatment alone [11, 12]. In the context of neoadjuvant chemotherapy (NACT), we assessed on-treatment response using ultrasound in the GeparTrio (G3) [13] and GeparQuinto (G5) [14] trials. In G3, we could demonstrate that response-guided switch of chemotherapy regimens can improve patient outcome.

During neoadjuvant chemotherapy (NACT), on-treatment samples can be utilized to uncover molecular mechanisms linked to therapy response, such as immune and proliferation signatures [15] and to pinpoint potential markers of resistance/response through comparative gene expression analysis between responders and non-responders [16].

Aim of this retrospective-prospective biomarker study was to evaluate the frequency of residual cancer cells in on-treatment samples from neoadjuvant clinical chemotherapy trials for BC, and to correlate their presence with response to treatment.

We also included the evaluation of two biomarkers: tumor-infiltrating lymphocytes and the proliferation marker Ki-67. Pre- and post-treatment levels of tumor-infiltrating lymphocytes (TILs) can predict response to chemotherapy and survival [17, 18] and chemotherapy may trigger or amplify a cytotoxic immune response [19]. High levels of the proliferation marker Ki-67 can predict a better response to neoadjuvant chemotherapy but also a poorer long-term prognosis due to more aggressive tumor biology [20]. During neoadjuvant aromatase inhibition, on-treatment Ki-67 evaluation also predicts patient outcomes [12]. We hypothesized that an increase in TILs or a decrease in Ki-67 could predict patient outcome after completion of chemotherapy.

## Methods

### Patients and samples

Patients were treated within the randomized, multi-center neoadjuvant clinical trials GeparQuattro (G4) [21–23], GeparQuinto (G5) [14, 24, 25] and GeparSixto (G6) [2]. Details on the study designs and outcomes are available in the original publications. In brief, G4 was a phase III study comparing the simultaneous or sequential use of capecitabine with epirubicin, cyclophosphamide and docetaxel (EC-T) with concomitant trastuzumab in HER2 + disease. G5 was a phase III study to evaluate EC-T with or without bevacizumab (B) in HER2-negative BC (setting I), to compare pCR rates of patients treated with paclitaxel with or without everolimus with HER2-negative BC without sonographic response after four cycles EC ± B (setting II) and to compare pCR rates in patients treated with EC-T followed by trastuzumab or lapatinib in HER2-positive disease (setting III). G6 was a phase II trial to evaluate the addition of carboplatin to neoadjuvant treatment for patients with triple-negative or HER2-positive BC. Biopsies were obtained at the time of diagnosis and during chemotherapy: in G4 and G5 after 4 of 8 cycles and in G6 after 2 of 6 cycles. Ultrasound was used to guide sampling on-treatment. Only biopsies from primary breast tumors were included in this study, not those from lymph nodes. Detailed information on sampling procedures (tumour size on ultrasound, number of biopsies, needle size) was not available for data analysis in these older clinical trials. All patients with available material in the central GBG tumor bank were eligible for this retrospective biomarker analysis. Of the 1495, 1948 and 588 patients in the G4, G5, and G6 trials, respectively, 106, 145 and 61 matched pre-therapeutic and on-treatment biopsies were available in the biobank and included in the study, resulting in 312 matched pairs. 15 samples had to be excluded due to insufficient pre-treatment material, resulting in a total of 297 matched samples. Table 1 details the baseline patient characteristics.

**Table 1:** Baseline characteristics of the study cohort

		All	G4	G5	G6
Subtype	HR−/HER2−	87 (29.3%)	19 (19%)	43 (30.9%)	25 (43.1%)
	HR+/HER2−	138 (46.5%)	53 (53%)	85 (61.2%)	0 (0%)
	HER2+	72 (24.2%)	28 (28%)	11 (7.9%)	33 (56.9%)
Response	no pCR	235 (79.1%)	81 (81%)	125 (89.9%)	29 (50%)
	pCR	62 (20.9%)	19 (19%)	14 (10.1%)	29 (50%)
On-treatment biopsy	tu+	207 (69.7%)	69 (69%)	112 (80.6%)	26 (44.8%)
	tu−	90 (30.3%)	31 (31%)	27 (19.4%)	32 (55.2%)
cT stage	T1	25 (8.4%)	0 (0%)	11 (7.9%)	14 (24.1%)
	T2	181 (60.9%)	68 (68%)	81 (58.3%)	32 (55.2%)
	T3	42 (14.1%)	16 (16%)	17 (12.2%)	9 (15.5%)
	T4	49 (16.5%)	16 (16%)	30 (21.6%)	3 (5.2%)
cN stage	N0	125 (42.1%)	43 (43%)	51 (36.7%)	31 (53.4%)
	N1-3	171 (57.6%)	57 (57%)	88 (63.3%)	26 (44.8%)
	NA	1 (0.3%)	0 (0%)	0 (0%)	1 (1.7%)
Grading	G1-2	156 (52.5%)	55 (55%)	76 (54.7%)	25 (43.1%)
	G3	136 (45.8%)	40 (40%)	63 (45.3%)	33 (56.9%)
	NA	5 (1.7%)	5 (5%)	0 (0%)	0 (0%)
Histology	NST	268 (90.2%)	89 (89%)	122 (87.8%)	57 (98.3%)
	Lobular	23 (7.7%)	9 (9%)	14 (10.1%)	0 (0%)
	Other	6 (2%)	2 (2%)	3 (2.2%)	1 (1.7%)
TILs Median = 8, IQR = 10	< 30% ≥ 30%	267 (89.9%) 30 (10.1%)	91 (91.0%) 9 (9.0%)	129 (92.8%) 10 (7.2%)	47 (81.0%) 11 (19.0%)
Ki-67 Median = 15.4, IQR = 22.7	≥ 15% < 15% NA	149 (50.2%) 142 (47.8%) 6 (2.0%)	55 (55.0%) 41 (41.0%) 4 (4.0%)	72 (51.8%) 65 (46.8%) 2 (1.4%)	15 (25.9%) 43 (74.1%) 0 (0%)

All patients provided written informed consent for participation in the study and the utilization of biomaterials for translational research purposes. The study protocol received approval from the relevant ethics committee and national competent authority.

### Biomarker analysis

An experienced pathologist (BVS, CD) reassessed the biopsies on an H&E-stained slide to identify the presence of invasive breast cancer (BC) at the German Breast Group’s central histopathology laboratory. On-treatment biopsies were categorized as positive for invasive tumor (tu+) if residual invasive cancer cells were observed. Ductal carcinoma in situ or other precursor lesions were not included in this classification.

The presence and quantity of tumor-infiltrating lymphocytes (TILs) in the stromal compartment were documented following the guidelines of the international TIL working group. This involved comparing the H&E-stained slide under review to standardized reference images. [26].

Immunostaining for Ki-67 was performed on a Ventana Discovery XT instrument (Ventana, Tucson, AZ) using the MIB-1 clone (diluted 1:50). Quantification of stained tumor cells was performed using a digital software solution (VMScope, Berlin, Germany) according to recommendation of the Ki-67 in BC working group [27]. For each case, three areas were chosen and counted, and the mean value of the different areas was used for analysis.

### Statistical considerations

Pathologic complete response (pCR) was defined as the absence of invasive or non-invasive BC in the breast and lymph nodes after completion of neoadjuvant treatment (ypT0 ypN0). Disease-free survival (DFS) was defined as the time from study entry to distant or local relapse or death from any cause.

Statistical analyses were computed in R 4.0.3 (R Project for Statistical Computing, RRID:SCR_001905). The change of TILs (Δ_TILs_) and Ki-67 (Δ_Ki-67_) was calculated as the difference between on-treatment and pre-treatment as a continuous parameter. To test the association of positive on-treatment biopsies (tu+) with tumor characteristics and pCR, chi-squared test was used. The Kaplan Meier method with log rank test was used to illustrate the association of response parameters with DFS. Uni- and bivariate Cox proportional hazard regression models were fit to examine the association of biomarkers with DFS. Logistic regression models were fit to examine the association of biomarkers with pCR.

## Results

### Frequencies of residual *cancer* cells and their association with patient and tumor characteristics (Fig. 1)

**Fig. 1:**
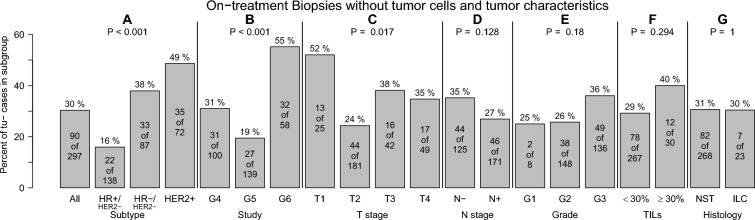
Frequency of on-treatment biopsies without tumor cells according to tumor characteristics. On-treatment biopsies without tumor cells were more frequent in triple-negative and HER2-positive breast cancers (**A**), in biopsies from G6 trial patients (**B**), and in smaller tumors (**C**). There was no statistical association with lymph node status (**D**), histological grading (**E**), tumor-infiltrating lymphocytes (**F**) or histology (**G**)

Residual cancer cells were present in biopsies of 207 patients (tu+; 70%) after 4 of 8 cycles (G4, G5) and 2 of 6 cycles chemotherapy (G6), respectively. 90 biopsies showed no residual disease (tu−; 30%). The highest frequencies of tu− biopsies were observed in patients with HER2+ (49%) and TNBC (38%) BC (Fig. 1). The highest frequency of tu− patients was observed among patients of the G6 trial (all patients had triple-negative or HER2-positive disease). The frequency of tu− patients was also higher in patients with small tumors. There was no statistically significant association with lymph node status, histological grading, TILs or histologic subtype (Fig. 1).

### Frequencies of residual *cancer* cells and their association with response to treatment (Figs. 2, 3)

**Fig. 2:**
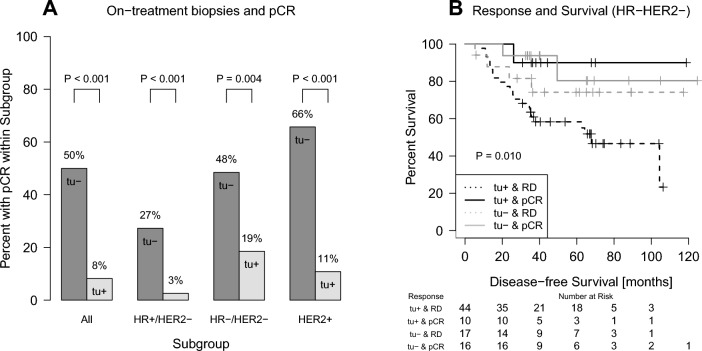
Association of cancer cells in on-treatment samples with pathological complete response after completion of chemotherapy (pCR,  **A**) and disease-free survival (**B**). The rate of pCR was 8% in patients with evidence of cancer cells on-treatment (tu+), but 50% when the biopsy was negative for cancer (tu−) (**A**). In the Kaplan–Meier analysis of patients with HR−/HER2− BC, residual cancer on-treatment (tu+) and residual disease (RD) after completion of chemotherapy were associated with shorter survival (**B**)

**Fig. 3:**
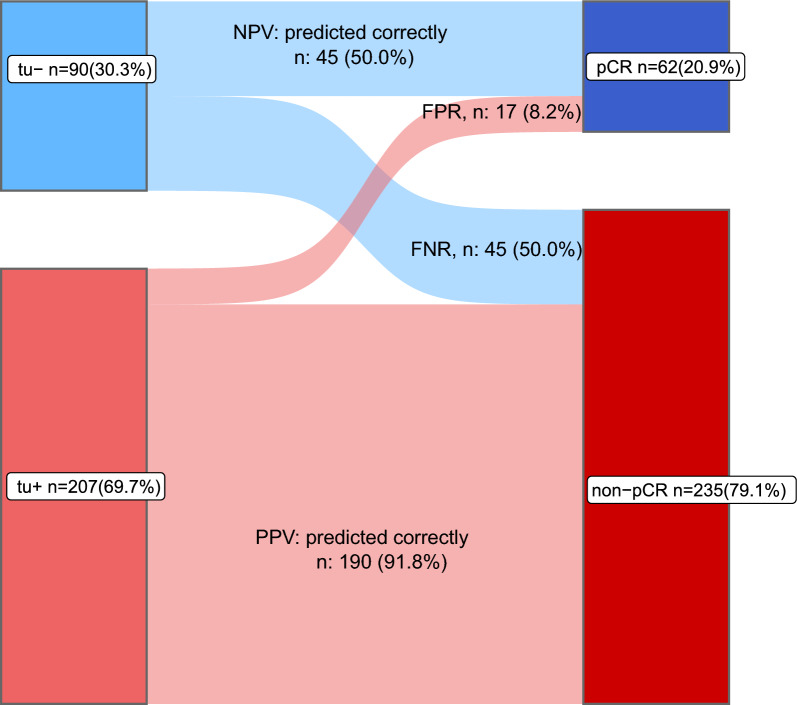
Distribution of patients with pCR or non-pCR after completion of chemotherapy according to on-treatment biopsies with (tu+) or without (tu−) residual cancer cells. Of the 90 on-treatment samples without residual cancer cells (blue), 50% had a pCR at the end of treatment and 50% had a non-pCR. Of the 207 on-treatment samples with residual cancer (red), 8.2% had a pCR and 91.8% had a non-pCR. NPV = negative predictive value; PPV = positive predictive value; FPR = false positive rate; FNR = false negative rate

In tu− patients a pCR was observed in 50% (45/90) (Fig. 2A). In contrast, only 17 of 207 (8%) patients with positive biopsies (tu+) had a pCR after completion of the full treatment course, and 92% had residual diseases (190/207). A similar association could be observed in the different BC subtypes (Fig. 2A). The distribution of patients with pCR or non-pCR after completion of chemotherapy according to on-treatment biopsies with (tu+) or without (tu−) residual cancer cells is demonstrated in a Sankey plot (Fig. 3) in detail. Sensitivity to predict residual disease was 0.81 (specificity 0.72). The positive and negative predictive values were 0.92 and 0.50, respectively.

In univariate Cox regression analyses, the absence of tumor cells in on-treatment biopsies was associated with a lower risk of relapse in patients with triple-negative disease (Table 2). However, the effect was not statistically significant when adjusted for pCR in a bivariate model (Table 3). The relationship between the presence of residual disease during and/or after chemotherapy and patient survival was demonstrated in a Kaplan–Meier analysis (Fig. 2B). Patients with residual cancer cells during chemotherapy (tu+) and residual disease (RD) after completion of the full course show the highest risk of relapse.

**Table 2:** Univariate Cox regression models to predict disease-free survival according to residual cancer in on-treatment biopsies during neoadjuvant chemotherapy

Subtype	Covariate	Hazard ratio (95% CI)	P
HR−/HER2−	tu− vs. tu+	0.402 (0.163–0.987)	0.047
HR+/HER2−	tu− vs. tu+	1.039 (0.428–2.525)	0.933
HER2+	tu− vs. tu+	1.77 (0.577–5.43)	0.318

**Table 3:** Bivariate Cox regression models to predict disease-free survival according to residual cancer in on-treatment biopsies during neoadjuvant chemotherapy and after treatment

Subtype	Covariate	Hazard ratio (95% CI)	P
HR−/HER2−	tu− vs. tu+	0.544 (0.217–1.369)	0.196
	pCR vs. no pCR	0.282 (0.083–0.964)	0.043
HR+/HER2−	tu− vs. tu+	1.14 (0.442–2.939)	0.787
	pCR vs. no pCR	0.689 (0.149–3.18)	0.633
HER2+	tu− vs. tu+	2.114 (0.589–7.584)	0.251
	pCR vs. no pCR	0.704 (0.197–2.516)	0.589

Patients with false negative predictions, i.e. those without residual tumor cells on-treatment but residual disease after completion of treatment were analyzed (Fig. S1). A higher frequency of false negative predictions was observed in HR+/HER2− disease. There was no statistically significant association with tumor stage, nodal status, grade or TILs. Patients with false positive predictions, i.e. those with residual cancer cells on-treatment, but with pCR after chemotherapy were demonstrated in Fig. S2. This was more frequently observed in HR−/HER2− cases, in the GeparSixto trial, in smaller tumors and in patients with negative clinical lymph node status.

### Dynamic change of TILs and Ki-67 and its association with patient outcome (Fig. 4)

**Fig. 4:**
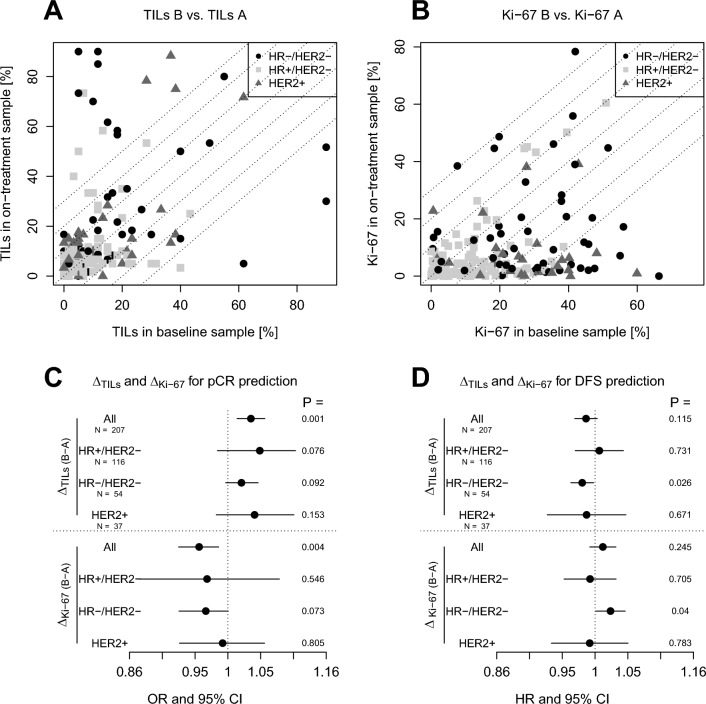
The number of tumor-infiltrating lymphocytes TILs (**A**) and Ki67 levels (**B**) in the on-treatment biopsies are plotted against the baseline sample. Only samples with residual cancer in the on-treatment biopsies are shown. The association of dynamic changes in TILs (ΔTILs) and Ki-67 (ΔKi-67) with pathological complete response (pCR) and disease-free survival (DFS) was evaluated using logistic regression and Cox regression analyses, respectively (**C**, **D**). An increase in TILs was associated with the likelihood of pCR across tumor subtypes (**C**) and with a lower risk of relapse in patients with triple-negative disease (**D**). No decrease in Ki-67 was associated with a lower likelihood of pCR across subtypes and a higher risk of relapse in patients with triple-negative disease. HR = hazard ratio, OR = odds ratio, CI = confidence interval

Paired TIL data was available for 207 patients (all samples with residual cancer on-treatment), paired Ki-67 data was available for 196 patients. There was no association between the dynamic change in Ki-67 and dynamic change of TILs (Fig. S3).

In patients with residual invasive cancer cells in the on-treatment biopsy an increase of TILs in a subset of patients was observed, while only a few patients showed a decrease (Fig. 4A). An increase of TILs was associated with a higher probability of pCR in the overall study cohort, but not within the BC subtypes (Fig. 4C). It was associated with a lower risk of relapse in patients with triple negative disease.

The proliferation index as measured by Ki-67 immunohistochemistry decreased in most patients during chemotherapy (Fig. 4B). The lack of decrease in Ki-67 was associated with a low probability of pCR in all patients and was associated with a higher risk of relapse in patients with triple negative disease (Fig. 4C).

In bivariate Cox regression analyses in patients with triple-negative disease adjusted for pCR, the dynamic change of TILs was statistically significantly associated with DFS. The change of Ki-67 was not significantly associated with relapse-free survival in a bivariate model adjusted for pCR (Table 4).

**Table 4:** Bivariate Cox regression models to predict disease-free survival according to pCR and dynamic change in TILs or Ki-67, respectively

	Covariate	Hazard ratio (95% CI)	*P*
HR−/HER2−	Delta TILs	0.979 (0.959–1.000)	0.048
	pCR vs. no pCR	0.190 (0.025–1.418)	0.105
HR−/HER2−	Delta Ki-67	1.019 (0.996–1.043)	0.098
	pCR vs. no pCR	0.211 (0.028–1.600)	0.132

## Discussion

In this retrospective research study, we analyzed on-treatment biopsies obtained during neoadjuvant chemotherapy (NACT) for breast cancer (BC).

30% of on-treatment biopsies showed no cancer cells. However, if residual cancer cells were detected, achieving a pathologic complete response (pCR) post-treatment was less likely. This suggests the potential for early treatment adjustment, including alternative chemotherapy agents or targeted therapies, to enhance response rates, particularly within the framework of clinical trials. In the GeparTrio and GeparQuinto neoadjuvant trials [13, 14], treatment was adjusted according to evaluation of on-treatment response using ultrasound and led to improved patient’s survival in GeparTrio. Biopsy procedures might be an additional tool to identify tumors prone to treatment failure early on treatment in future trials.

If no cancer cells were detected during treatment, this information could not reliably predict chemotherapy outcome, as the rate of pCR was 50% in this group in the current study. This suggests that sampling error of the residual disease may have led to a false negative on-treatment sample. False negative prediction was more frequent in HR+/HER2− disease, reflecting a tumor biology with a lower a priori probability for pCR. False positive prediction (cancer cells on-treatment but pCR after therapy) was more frequent in HR−/HER2− tumors, in the GeparSixto trial and in smaller tumors, reflecting patients with a higher a priori probability of pCR.

As our study collected on-treatment samples for translational research rather than for predicting pCR or guiding treatment decisions, its comparability to studies focused on on-treatment pCR prediction may be limited (reviewed in [6]). The reported negative predictive values of these studies vary, ranging from 71% for core needle biopsy (CNB) to as high as 95% for vacuum-assisted biopsy, which offers a larger specimen for analysis. However, with its false negative rates reaching 49.3%, presents a considerable margin for error [28, 29]. However, these investigations aimed to evaluate pathologic complete response (pCR) after completion of neoadjuvant chemotherapy (NACT) before surgery, utilizing minimally invasive methods to identify patients potentially eligible for surgery omission. These trials did not assess the capability to predict pCR early during treatment.

In a report of 40 patients from the ISPY2 trial, the presence of cancer cells in mid-treatment biopsies was associated with a 20% pCR rate, while the pCR rate in patients without invasive cancer cells was 90% [30].

Regarding survival, patients with triple-negative tumors and residual disease both on- and post-treatment had the highest risk of relapse. Patients without the evidence of cancer cells on-treatment but residual disease after the completion of the full course of treatment had a lower risk. This observation could be explained by the fact that the biopsy procedure is more likely to miss smaller tumors or tumors with only a minimal amount of residual disease.

Pre-treatment levels of TILs can be used to predict response to chemotherapy and patient’s survival in BC [17]. Chemotherapy might induce or augment a cytotoxic immune response [19] and an influx of TILs during treatment is associated with better response [31]. Moreover, their presence in surgical specimens is associated with better outcome [18] and a gene signature for prediction of TILs in post-treatment samples is predictive of survival [32].

In this study, we observed an increase of TILs in a subset of patients and only a few cases showed a decrease. An increase of TILs was associated with a higher probability of pCR in all patients and with a lower risk of relapse in patients with TNBC reflecting their known predictive value in triple-negative disease. This observation is particularly interesting, as it can identify patients early on treatment that still harbor invasive tumor residuals, where continuing standard therapy might be the option that is superior to a change in treatment plan. Further investigation in this group of patients could help to refine adapting tumor response into treatment plans and could ultimately allow to move the risk assessment after NACT to an earlier timepoint.

The marker of tumor cell proliferation Ki-67 can be used to predict response to NACT and patient’s survival [20, 33]. High levels are typically associated with better response to cytotoxic treatment but shorter long-term outcome due to more aggressive tumor biology. In the context of neoadjuvant aromatase inhibition, on-treatment evaluation of Ki-67 can predict patient outcome [11, 12].

In this study, most patients showed a decrease in Ki-67 index and this was associated with a higher probability of response in triple-negative disease. It was also associated with a lower risk of relapse in triple negative disease, but not in other subtypes. Bivariate survival analyses demonstrated that these effects were probably due to the association with pCR and its strong association with survival in this subtype.

From a translational research perspective, these observations suggest that on-treatment biopsies could be valuable for studying mechanisms of therapy resistance and predicting failure to achieve pathologic complete response (pCR) after neoadjuvant chemotherapy (NACT). However, they are not suitable for identifying markers of chemotherapy sensitivity, as highly sensitive tumors would not contain residual cancer cells in on-treatment biopsies. To address this, further analyses should focus on revisiting naive biopsies from patients who achieved early pCR. On a molecular level, NACT induces global changes in gene expression [34]. Examples of alterations are genes involved in proliferation, epithelial-mesenchymal transition and metabolic processes [35]. Analysis of serial biopsy samples during chemotherapy allows the characterization of mechanisms of early response and adaption to therapy. In such a study, a decreased expression of genes related to immune response and proliferation could be observed and the downregulation of cell-cycle inhibitors was associated with worse response [15]. The use of on-treatment biopsies for patient stratification should be further explored in the context to clinical trials and should be a part of the study protocol.

Several limitations of the study warrant consideration. Firstly, it's important to note that the biopsy procedure was not strictly standardized according to the study protocol. Detailed records of the sampling procedure were not available in the database of these older clinical trials. This limits the interpretation of the results, as bias due to patient selection (e.g. due to different or absent tumor visibility on ultrasound) or different sampling procedures (e.g. different number and/or diameter of biopsies) cannot be ruled out. At the time of the clinical trials used in this study, no clips were placed in the primary tumor area prior to treatment. The accuracy of the biopsy procedure may be limited. Additionally, variations in the timing of the biopsy procedure across the three trials were inevitable due to differences in study design. In GeparQuattro and GeparQuinto, on-treatment samples were obtained after 4 of 8 cycles, in GeparSixto after 2 of 6 cycles. Moreover, GeparQuattro and GeparQuinto switched from anthracycline to taxane therapy following the biopsy, whereas GeparSixto continued with the same regimen (concurrent taxane/anthracycline). The collection of on-treatment biopsies was not a mandatory part of the study protocol with a potential selection bias and comparably small samples sizes in subgroup analyses. The study was primarily designed to collect material for translational research purposes. It must be considered that patients within this trial had been treated before 2012 which possibly limits accuracy of on-treatment biopsy due to less experienced examiners and less refined examination instruments. Regimen not matching current standards thereby limiting the chances of achieving a pCR, are also able to influence false negative rates and predictive values of on treatment biopsies. Immune checkpoint inhibitors were not part of neoadjuvant chemotherapy in patients with triple-negative breast cancer, and patients with HER2-positive disease did not receive dual blockade.

In summary, our findings show that on-treatment biopsies can effectively predict non-pCR across breast cancer (BC) subtypes when residual cancer is present. This discovery presents potential avenues for tailoring therapy concepts in future clinical trials, such as implementing de-escalation strategies for responders and exploring experimental treatments for non-responders.

A reliable method for predicting chemotherapy response during treatment could greatly impact patient management and improve treatment strategies. By monitoring response, treatment plans can be tailored to individual patients, and if a patient is unlikely to respond well to the initial regimen, adjustments can be made to optimize therapeutic outcomes by switching to more effective drugs or modifying the dose. Early prediction of non-response allows for discontinuing ineffective treatments, avoiding unnecessary toxicity and complications, and better allocation of healthcare resources.

For example, ISPY2 trial is evaluating a combination of mid-treatment MRI and core biopsies to predict response and guide de-escalation of neoadjuvant treatment [36].

Predicting response helps plan the extent of surgery more accurately, potentially reducing the need for extensive procedures like mastectomy. Early prediction of treatment success can improve overall prognostication and support informed decision-making for post-neoadjuvant treatment and observation strategies.

Moreover, analyzing sequential biopsies could be instrumental in identifying molecular markers of therapy resistance to enhance our understanding of tumor biology and its adaptation to therapy. These advancements can ultimately improve overall treatment paradigms, improving the precision and effectiveness, leading to better patient outcomes. Further research is warranted to determine whether more standardized or extensive sampling procedures, or their combination with additional clinicopathological features, could enhance the sensitivity of on-treatment biopsy procedures.

## Supplementary Information

Supplementary Material 1: Figure S1. Comparison of tumors with false negative predictions (no residual cancer on-treatment but non-pCR after surgery) with tumors with true negative predictions. False negative predictions were more frequent in HR+/HER2− disease. (A). There was no statistically significant association with the clinical trial (B), tumor stage (C), nodal status (D), histological subtype (E) or tumor infiltrating lymphocytes (F).

Supplementary Material 2: Figure S2. Comparison of tumors with false positive predictions (residual cancer on-treatment but pCR after treatment) with tumors with true positive predictions. False positive predictions were more frequent in HR−/HER2− disease (A), in the GeparSixto trial (B), smaller tumors (C) and in tumors with negative lymph node status (D). There was no association grade (E) or tumor infiltrating lymphocytes (F).

Supplementary Material 3: Figure S3. The change in Ki-67 between the two time points is plotted against the change in tumor-infiltrating lymphocytes. There was no association between the two.

## Data Availability

All data needed for this analysis and results generation is included in this published article.

## Abbreviations

B: Bevacizumab
CI: Confidence interval
DFS: Disease-free survival
EC-T: Epirubicin, cyclophosphamide and docetaxel
G4: GeparQuattro
G5: GeparQuinto
G6: GeparSixto
HER2: Human epidermal growth factor receptor
HR: Hazard ratio
NACT: Neoadjuvant chemotherapy
pCR: Pathological complete response
RD: Residual disease
TILs: Tumor-infiltrating lymphocytes
TNBC: Triple negative breast cancer
tu+: Residual tumor
tu−: No residual tumor
