# Supplementary figures and images for: On-treatment biopsies to predict response to neoadjuvant chemotherapy for breast cancer
Source: Breast Cancer Res. 2024 Sep 24;26:138. doi: 10.1186/s13058-024-01883-w (PMC11423510; doi:10.1186/s13058-024-01883-w)

**A**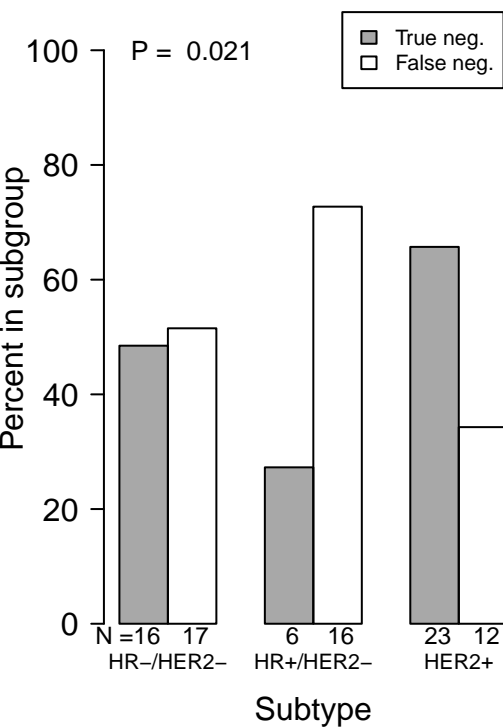**B**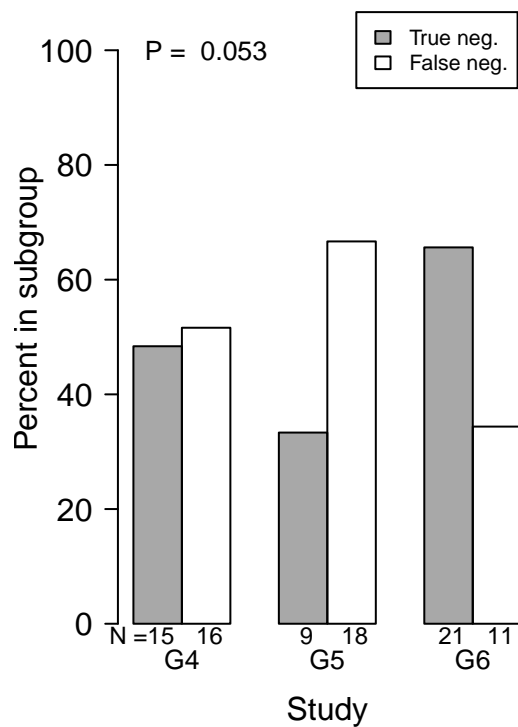**C**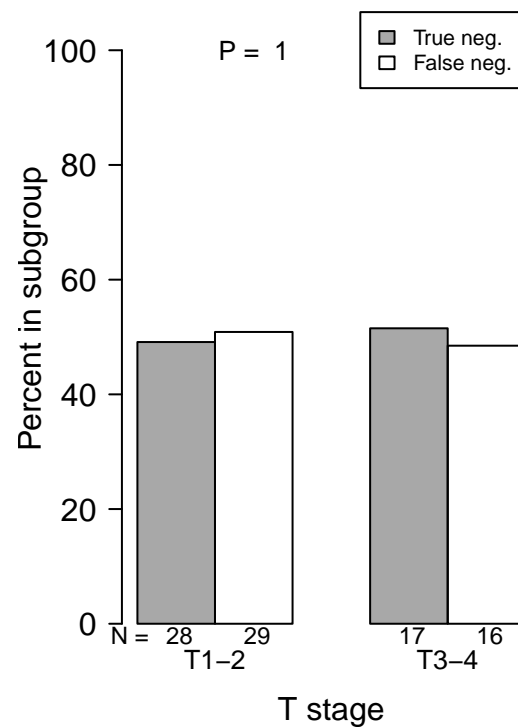**D**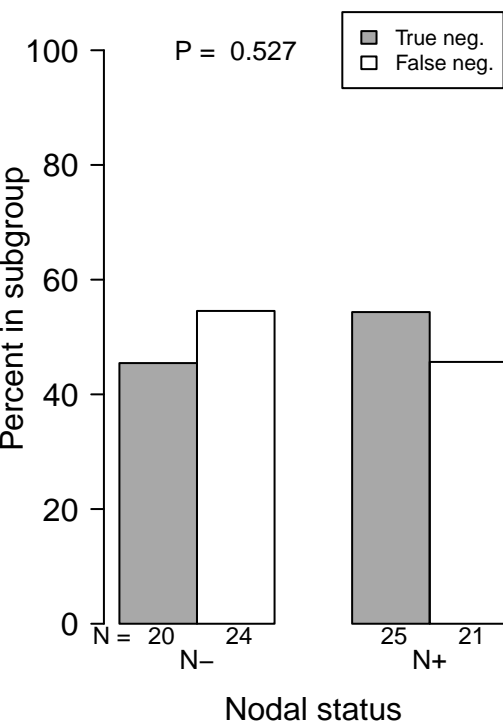**E**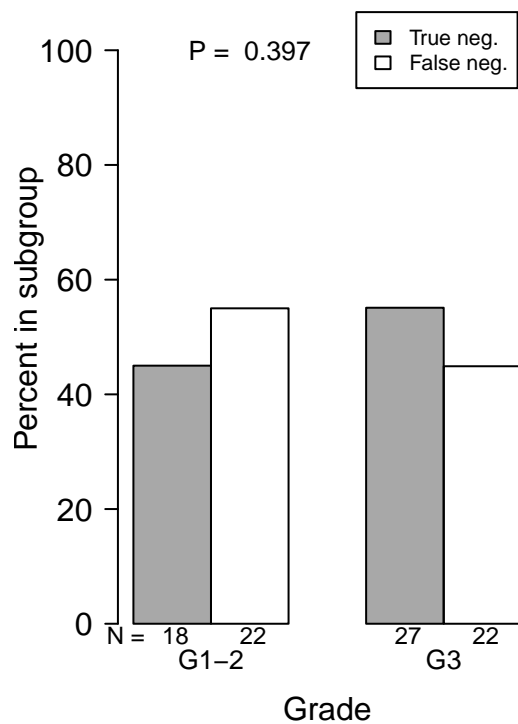**F**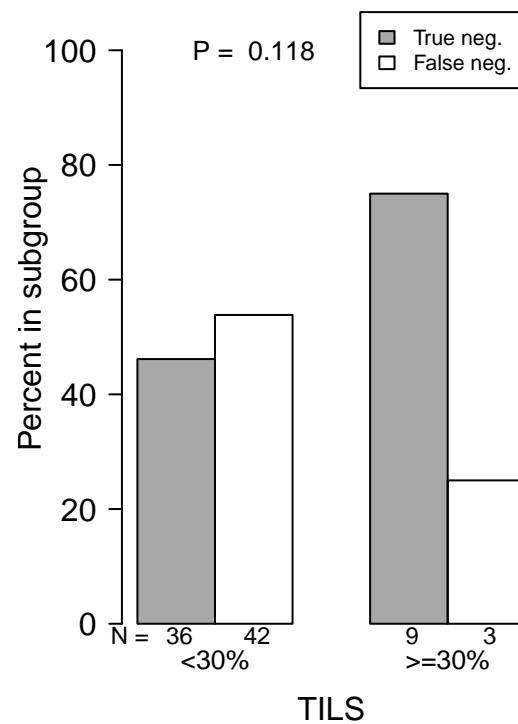

Supplement: Supplementary file 1 — Supplementary Material 1: Figure S1. Comparison of tumors with false negative predictions (no residual cancer on-treatment but non-pCR after surgery) with tumors with true negative predictions. False negative predictions were more frequent in HR+/HER2− disease. (A). There was no statistically significant association with the clinical trial (B), tumor stage (C), nodal status (D), histological subtype (E) or tumor infiltrating lymphocytes (F). [file 13058_2024_1883_MOESM1_ESM.pdf]

**A**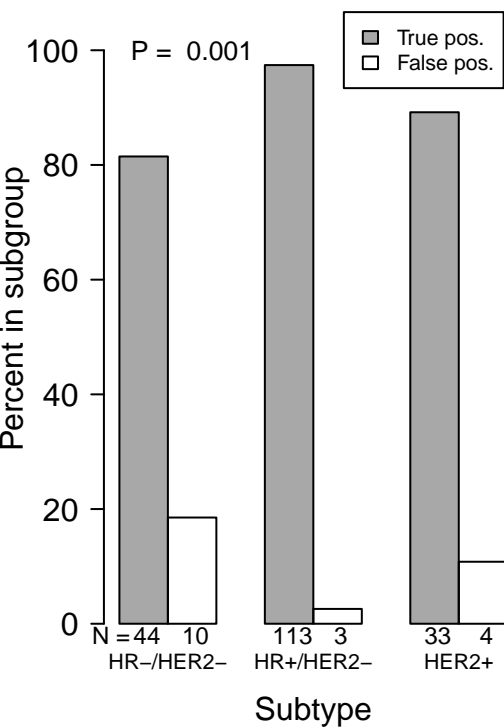**B**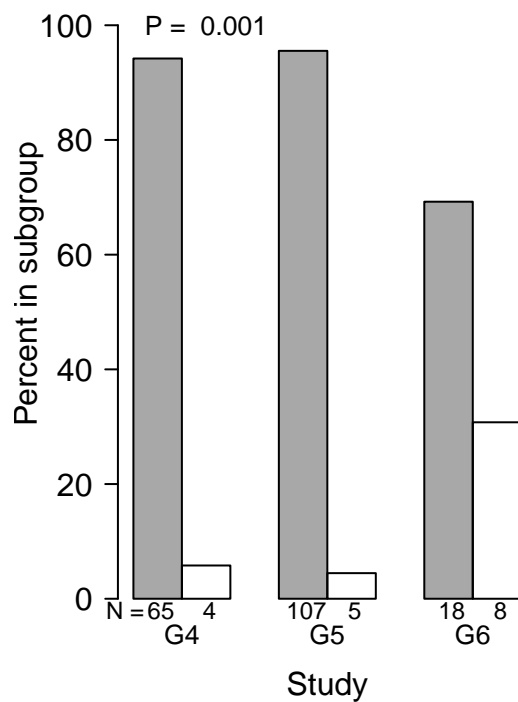**C**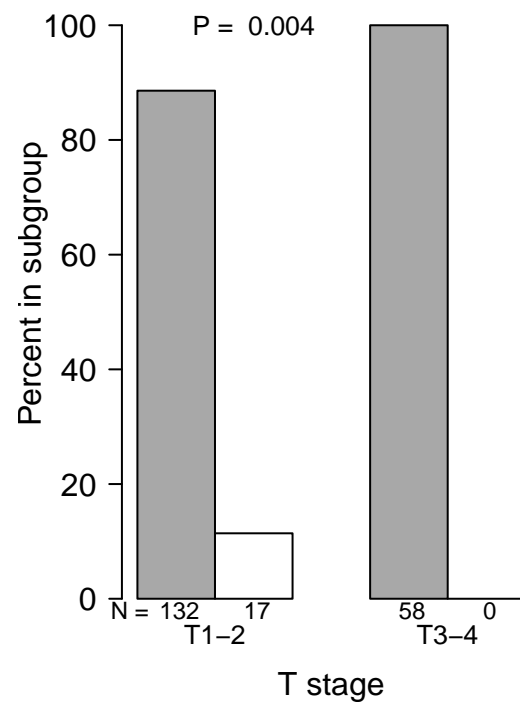**D**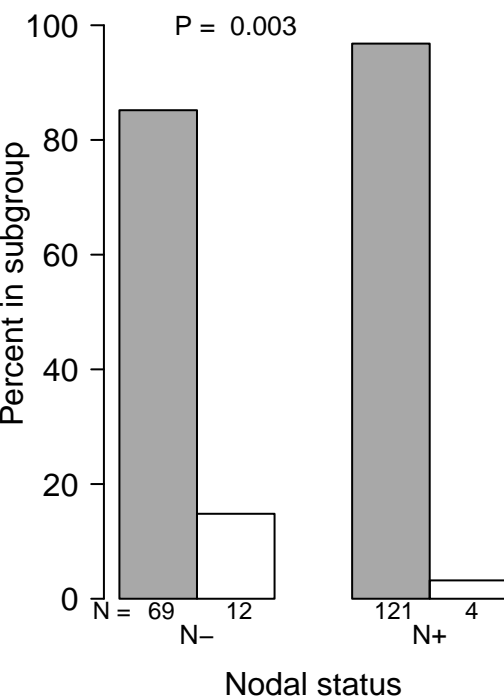**E**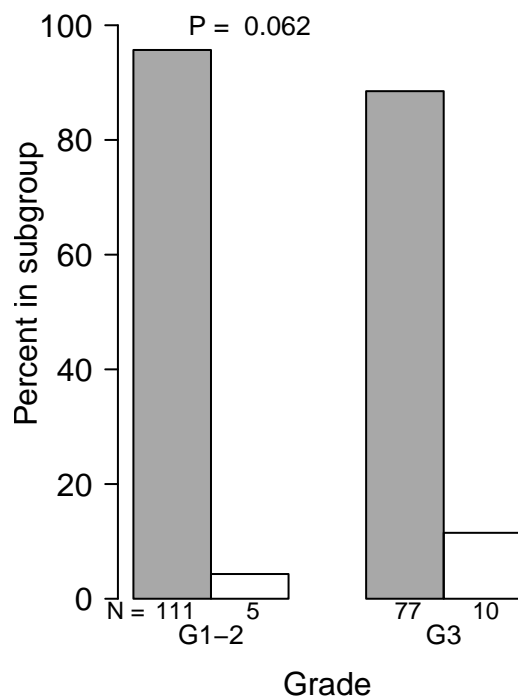**F**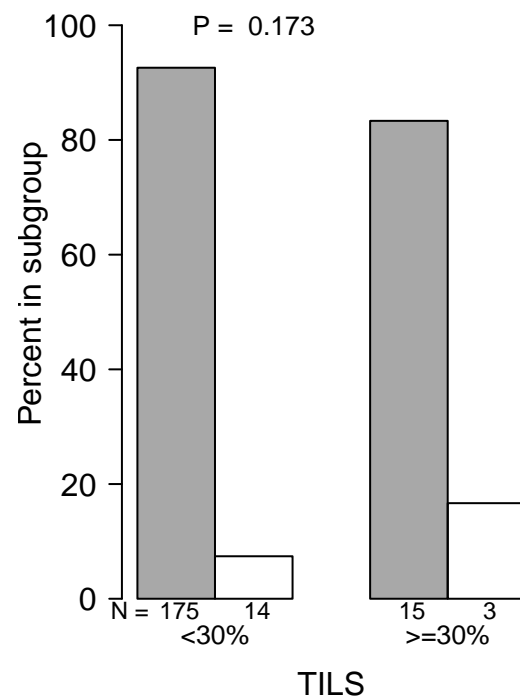

Supplement: Supplementary file 2 — Supplementary Material 2: Figure S2. Comparison of tumors with false positive predictions (residual cancer on-treatment but pCR after treatment) with tumors with true positive predictions. False positive predictions were more frequent in HR−/HER2− disease (A), in the GeparSixto trial (B), smaller tumors (C) and in tumors with negative lymph node status (D). There was no association grade (E) or tumor infiltrating lymphocytes (F). [file 13058_2024_1883_MOESM2_ESM.pdf]

$\Delta_{\text{Ki-67}}$  vs.  $\Delta_{\text{TILs}}$

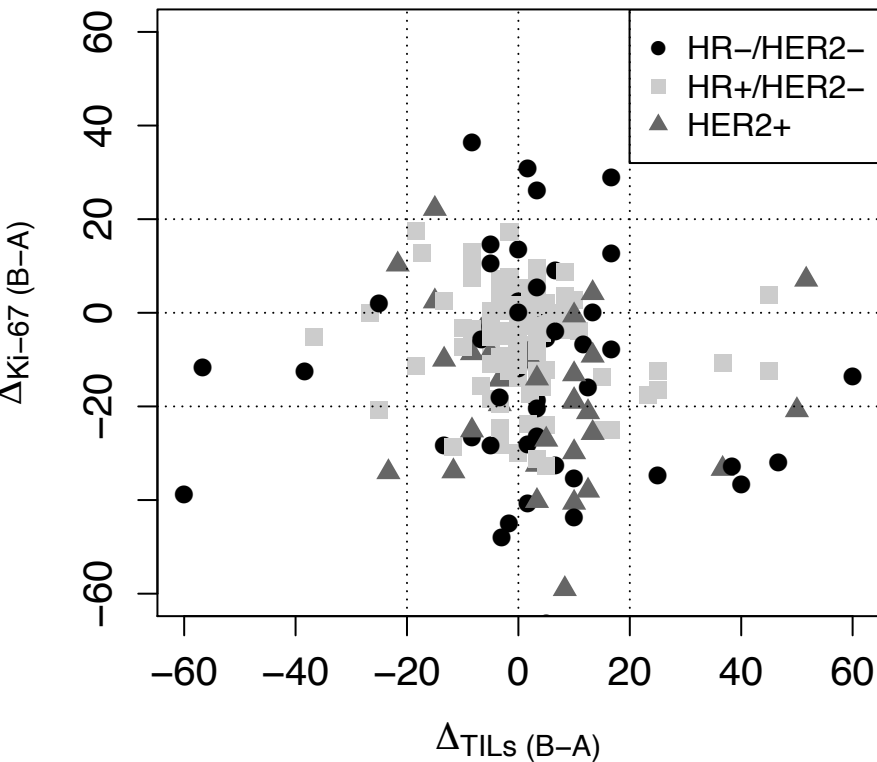

Supplement: Supplementary file 3 — Supplementary Material 3: Figure S3. The change in Ki-67 between the two time points is plotted against the change in tumor-infiltrating lymphocytes. There was no association between the two. [file 13058_2024_1883_MOESM3_ESM.pdf]
